# Preparatory Medical School

**Published:** 1858-07

**Authors:** 


### Preparatory Medical School.

The Faculty of the Atlanta Medical College, have determined to establish a *Preparatory Medical School*, in connection with this Institution. It will commence operations on the 1st Monday of November, and continue until the last of February, and will consist of Systematic Lectures (of a more elementary character than those of theregular course)—Examinations; and facilities for acquiring a knowledge of practical medicine, afforded by Hospital and Dispensary practice. The *Prepara-*

*tory* course will be conducted exclusively by the Faculty, for which a specific charge will be made, to be deducted, however, from the fees of the regular course. So that it will be perceived that the Atlanta Medical College, will hereafter give *eight months* instruction in medicine *annually*, for the same fees which they have heretofore charged for the usual term of four months. It is of course to be distinctly understood, that *this has no connection with the requisitions for graduation*, and will not be recognized as a *Regular* course of lectures.

The object and intention of the Atlanta Medical College, is to offer the best advantages for the pursuit of medical science, and will not be behind in the effort to compete with other Institutions in furnishing facilities for instruction—it is to this mode of competition, that she looks to success, and not to the dishonorable means of detraction and slander.

The announcement containing the details of the proposed *Preparatory* School of Medicine, will shortly be issued. For further information application may be made to the Dean of the Atlanta Medical College.

